# Posterior epidural migration of lumbar intervertebral fragment: case report

**DOI:** 10.11604/pamj.2015.21.80.6993

**Published:** 2015-06-02

**Authors:** Soueilem Mohamed Bouya, Ben Ousmanou Djoubairou, Naama Okacha, Miloudi Gazzaz, Brahim El Mostarchid

**Affiliations:** 1 Department of Neurosurgery, Mohammed V Military Teaching Hospital, Mohammed V University, School of Medicine, Hay Riyad, 1018 Rabat, Morocco

**Keywords:** Posterior epidural disk fragment, magnetic resonance imaging, surgery

## Abstract

Disc fragments are well known to migrate to superior, inferior, or lateral sites in the anterior epidural space, posterior epidural migrated lumbar disc fragments is an extremely rare disorder. Posterior epidural migrated lumbar disc fragments are often confused with other posterior epidural space-occupying lesions (cysts, abscesses, tumors, and hematomas). We reported the case of a 52- year-old man presented with progressive not systematizes bilateral radiculopathy complicated one week before admission a difficulty dorsiflexion prevents the start, and the stared to use crutches. Clinical examination revealed steppage gait and a strength score of 3/5 on dorsiflexion of feet. MR imaging of lumbar spine showed right posterolateral epidural mass that compressed the dural sac at the L3-4 level. Patient underwent surgery using posterior approach, an L3 laminectomy was performed, the extruded disk fragment was gently removed and L3-L4 interspace was explored. Histopathology confirmed the (PEMLIF). Postoperative course was uneventful.

## Introduction

The migration of the lumbar intervetebral disc fragment to the posterior epidural space (PEMLIF) is a rare event just few cases are reported in English literature [1]. Clinical presentation is indistinguishable from that of a typical lumbar disc herniation (LDH) [1, 2]. Magnetic Resonance imaging (MRI) findings can help to increase the preoperative diagnosis of PEMLIF but the appearance of these findings remains inconsistent [2]. Treatment consisted of removal of the extracted fragment through hemi- or complete laminectomy, physiotherapy in post operative course promotes good recovery. However in patient without neurological deficit, neurologic symptoms worsen over time or acute cauda equina syndrome, conservative management including the oral administration of nonsteroidal anti-inflammatory drugs (NSAIDs) and a caudal block, were confirmed to have achieved a complete recovery.

## Patient and observation

**Figure 1:**
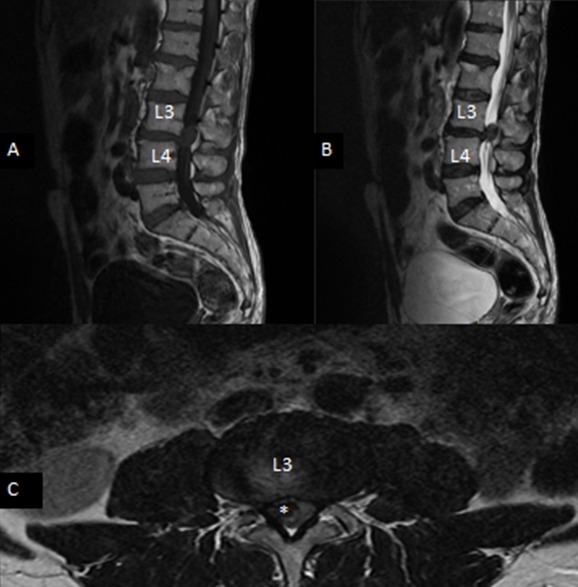
Sagittal T1 (A), sagittal T2 (B), and axial (C) T1-weighted MR images revealing a right posterolateral epidural mass (star) that compressed the dural sac at the L3-4 level

**Figure 2:**
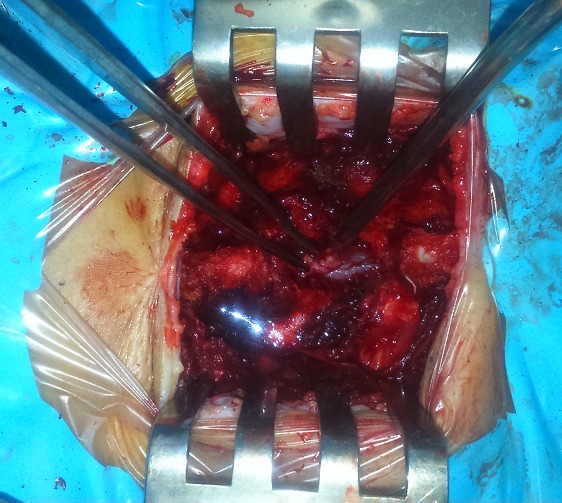
Intraoperative photograph of the dural sac after L3 laminectomy showed Extracted disk fragment (pliers) with yellowish appearance and attachment to the posterior dural sac

A 52-year-old man presented with 2 years history of intermitent lumbago. Since 2 months ago, the experienced a progressive not systematizes bilateral radiculopathy complicated one week before admission a difficulty dorsiflexion prevents the start, and the stared to use crutches. Clinical examination revealed steppage gait and a strength score of 3/5 on dorsiflexion of feet, patellar and Achilles reflex were depressed, and there were any sphincter symptoms or saddle hypoanesthesia. Past history revealed no history of trauma neither fever, nor weight loss. Laboratory test was also normal. Plain radiographs of the lumbar sacral spine were essentially normal, MR imaging of lumbar spine was realized based emergency and objectified a reshuffle osteoarthritis associated with degenerative disc disease and right posterolateral epidural mass that compressed the dural sac at the L3-4 level (Figure 1). Patient underwent surgery using posterior approach, an L3 laminectomy was performed, after removal of the ligamentum flavum, the extruded disk fragment embedded in fibrous epidural tissue was readily visible posterior and lateral to the thecal sac. The fragment clearly compressed and was adherent to the thecal sac (Figure 2). The fragment was gently removed and L3-L4 interspace was explored. Rupture at the posterior longitudinal ligament (PLL) was detected, and an L3-L4 diskectomy was performed. Histopathology confirmed the (PEMLIF). Postoperative course was uneventful; after 2 months of physiotherapy patient completely relieved of his legs weakness.

## Discussion

Vicenzo Lombarti the first author to publish two cases of PEMLIF in 1973 [3] described the lesion as “a posterior rotation of annulus fibrosus”. It most often migrated in the anterior epidural space, rostral, caudal and lateral migrations are the most clinically important modes of this migration [1, 4, 5]. Posterior migration of the free fragments causing cauda equina syndrome (CES), is exceptionally rare [6, 7]. The disk fragment migration patterns are generally limited by the attachments of the PLL and its associated midline septum and peridural or lateral membrane and the nerve root itself [1, 4, 6, 7]. The etiopathogenic mechanism of PEMLIF remains unclear, some theories have been advanced. Several authors have attributed these lesions to the presence of anatomical barriers that prevent disk fragment migration [1, 4, 5], a problem with any one of these barriers may facilitate PEMLIF, which may be greater when the angle formed by the nerve root and the dural sac is obtuse, in this condition the nerve root is not in its supportive position as a potential key anatomical barriers to PEMLIF [6–8]. The PEMLIF may be expected to present clinically with isolated acute or chronic lumbago to significant neurologic symptoms, to the extent of presenting as CES [4], higt lumbar levels are more often affected [1]. Our patient presented with progressive not systematizes bilateral radiculopathy complicated one week before admission a difficulty dorsiflexion of feet with strength score of 3/5. Definitive diagnosis of PEMLIF may be difficult. Conventional axial and sagittal magnetic resonance imaging has been the method of choice for radiologic diagnosis of lumbar degenerative conditions. However, it may not always absolutely differentiate similar processes, such as herniated disk, epidural hematoma, and abscess [9]. Most of the time magnetic resonance images may mimic those of other more common posterior epidural lesions such as abscess, hematoma, and malignancy, although the ring enhancement after gadolinium administration is typical [1, 2]. Herniated disks are usually hypointense on T1-weighted images and hyperintense on 80% of T2-weighted images [1, 6, 9]. Making a diagnosis between posterior epidural migrated lumbar disc fragments and hematomas is very difficult. Disc herniation with migration may retain contact with the disc space from which they arose, whereas hematomas can be distinguished from disc fragments by the lack of continuity with a disc space [10]. In case of lesion enhancement after injection of gadolinium or suspicion of epidural abscess dosage of infectious laboratory markers would be systematic (C- reactive protein, erythrocyte sedimentation rate). Early surgery should be the first choice to prevent severs neurologic deficits [1, 4–8]; generally surgical results for PEMLIF are encouraging. All patients experienced a full recovery from their lumbar radicular pain, leg weakness, and/or sphincter disturbance [1]. The surgical outcome of CES caused by PEMLIF appears better than that of symptoms caused by conventional LDH [1, 4, 6]. Since 1973, 61 cases of PEMLIF have been reported including our present report; only 3 cases were treated using conservative management (NSAIDs, caudal block). The treatment for posterior epidural migrated lumbar disc fragments should therefore be determined based on the severity and course of the patient's symptoms.

## Conclusion

Despite their rarity PEMLIF should be raised before any CES, or isolated neurological deficit of lower limbs resent or old, to realize an MRI emergency is to propose if appropriate, a precocious and radical surgical treatment.
